# Temporal dynamics of *Mycobacterium tuberculosis* genotypes in New South Wales, Australia

**DOI:** 10.1186/1471-2334-14-455

**Published:** 2014-08-23

**Authors:** Ulziijargal Gurjav, Peter Jelfs, Nadine McCallum, Ben J Marais, Vitali Sintchenko

**Affiliations:** Sydney Medical School and the Marie Bashir Institute for Infectious Diseases and Biosecurity, The University of Sydney, Sydney, Australia; NSW Mycobacterium Reference Laboratory, Centre for Infectious Diseases and Microbiology Laboratory Services, Institute of Clinical Pathology and Medical Research – Pathology West, Sydney, Australia; Centre for Infectious Diseases and Microbiology – Public Health, Westmead Hospital, Sydney, Australia

**Keywords:** *Mycobacterium tuberculosis*, Population structure, Molecular epidemiology, Cluster analysis

## Abstract

**Background:**

Molecular epidemiology of *Mycobacterium tuberculosis,* its transmission dynamics and population structure have become important determinants of targeted tuberculosis control programs. Here we describe recent changes in the distribution of *M. tuberculosis* genotypes in New South Wales (NSW), Australia and compared strain types with drug resistance, site of disease and demographic data.

**Methods:**

We evaluated all culture-confirmed newly identified tuberculosis cases in NSW, Australia, from 2010-2012. *M. tuberculosis* population structure and clustering rates were assessed using 24-loci Mycobacterial interspersed repetitive unit (MIRU) analysis and compared to MIRU data from 2006-2008.

**Results:**

Of 1177 tuberculosis cases, 1128 (95.8%) were successfully typed. Beijing and East African Indian (EAI) lineage strains were most common (27.6% and 28.5%, respectively) with EAI strains increasing in relative abundance from 11.8% in 2006-2008 to 28.5% in 2010-2012. Few cases of multi-drug resistant tuberculosis were identified (18; 1.7%). Compared to 12-loci, 24-loci MIRU provided improved cluster resolution with 695 (61.6%) and 227 (20.1%) clustered cases identified, respectively. Detailed analysis of the largest cluster identified (an 11 member Beijing cluster) revealed wide geographic diversity in the absence of documented social contact.

**Conclusions:**

EAI strains of *M. tuberculosis* recently overtook Beijing family as a prevalent cause of tuberculosis in NSW, Australia. This lineage appeared to be less commonly related to multi-drug resistant tuberculosis as compared to Beijing strain lineage. The resolution provided by 24-loci MIRU typing was insufficient for reliable assessment of transmissions, especially of Beijing family strains.

**Electronic supplementary material:**

The online version of this article (doi:10.1186/1471-2334-14-455) contains supplementary material, which is available to authorized users.

## Background

Globally, tuberculosis remains a major cause of disease and untimely death. In 2011, there were an estimated 8.8 million incident cases and 1.8 million deaths attributed to tuberculosis [1]. Although the global tuberculosis incidence rate has been declining at ~2% per year since 2002, the impact of control efforts remains limited in areas affected by poverty, human immunodeficiency virus infection or drug resistance in *Mycobacterium tuberculosis*[1, 2]. In non-endemic areas such as Australia disease rates remain low, but progress towards tuberculosis elimination is limited. In fact, the rate of bacteriologically confirmed tuberculosis in Australia has gradually risen from 3.7/100,000 in 1998 to 4.9/100,000 in 2009 [3].

The state of New South Wales (NSW) report the highest absolute number of tuberculosis cases within Australia, but disease rates are highly variable [4]. NSW surveillance data indicate that the majority of tuberculosis cases identified occur among recent immigrants who acquired *M. tuberculosis* infection in their country of origin [5]. Tuberculosis incidence rates in excess of 60/100,000 in parts of metropolitan Sydney represent concentrated pockets of imported disease, which may support local transmission [6] .The NSW Mycobacterial Reference Laboratory (MRL) provides ongoing laboratory surveillance to help identify local tuberculosis outbreaks and guide public health responses. Since 2006, the laboratory performed routine strain typing using mycobacterial interspersed repetitive unit (MIRU) analysis to describe the *M. tuberculosis* population structure and detect local transmission events. MIRU typing identifies variable number tandem repeats found in 41 loci across the *M. tuberculosis* genome [7]. Its discriminatory power varies depending on the *M. tuberculosis* population structure and the number of loci used, for example 24-loci MIRU is more discriminatory than 12 and 15-loci MIRU [8, 9].

An assessment of new tuberculosis cases notified in NSW from 2009-2011 indicated that 79.7% of cases were immigrants born in tuberculosis endemic countries; drug resistant disease was rare. [5]. The use of 12-loci MIRU typing described the *M. tuberculosis* population structure, but provided insufficient discrimination to confidently identify local transmission chains. The current study aimed to examine temporal trends in the *M. tuberculosis* epidemiology and drug resistance rates. In addition, we compared the discriminatory power of 24- and 12-loci MIRU in a setting where the majority of *M. tuberculosis* strains are imported from Asia.

## Methods

### Study setting and design

We report data from ongoing prospective surveillance conducted by the NSW MRL at the Institute of Clinical Pathology and Medical Research (ICPMR) in Sydney, Australia. It receives *M. tuberculosis* isolates from tuberculosis cases diagnosed throughout the state. New culture confirmed tuberculosis cases identified between January 2010 and December 2012 were included in the study. Basic patient demographic data including age, gender, residential postcode and site of disease were retrieved from the ICPMR information system. The International Classification of Disease (ICD-9) coding was used to assess the site of disease, among those diagnosed with tuberculosis (category 9) subcategory 010, 011 and 012 were classified as respiratory disease and all other subcategories regarded as non-respiratory disease. Duplicate isolates and patients infected with mycobacterial species other than *M. tuberculosis* complex were excluded from the analysis.

### Isolates

All *M. tuberculosis* isolates were identified by conventional methods and their identity was confirmed by high-performance liquid chromatography (HPLC) of mycolic acids (Waters™ LS Module 1Plus, Milford, MA, USA), DNA probes and in-house PCR, targeting the 16S-23S rRNA gene internal transcribed spacer region, when necessary. Susceptibilities to isoniazid (INH) and rifampicin (RIF), pyrazinamide and ethambutol were tested at “breakpoint” concentrations using the BACTEC MGIT™ 960 system (Becton Dickinson) according to the manufacturer’s instructions. Isolates were considered resistant to INH and RIF when they grew in the presence of 0.4 mg/L and 0.1mg/L of drug, respectively.

### Genotyping and mapping

Isolates were genotyped using 24-loci MIRU as previously described [8]. *M. tuberculosis* population structure and drug resistance profiles were compared for the time period covered by the current study (2010-2012) and a previous assessment done from 2006-2008 [10]. For cluster analysis, two or more strains with identical MIRU profiles were considered a cluster. The recent transmission rate was calculated as follows [(number of clustered isolates - number of clusters)/total number of cultured isolates] [11]. *M. tuberculosis* lineage assignment was done using the on-line MIRU-VNTR*plus* database (http://www.miru-vntrplus.org). The isolates were assigned to global lineages and sublineages: Lineage 1 – Indo-Oceanic (East-African-Indian sublineage); Lineage 2 – East-Asian (Beijing sublineage); Lineage 3 – East-African-Indian (Delhi/Central Asian sublineage) and Lineage 4 – Euro-American (Latin-American and Haarlem sublineages). Tuberculosis cases were geomapped using their postcodes of residence and the Australian Pathogen Intelligence Community Space (APICS) online tool (http://www.abin.org.au); 11 cases belonging to the largest single cluster identified by 24-loci MIRU typing were mapped.

### Statistical analysis

Associations between *M. tuberculosis* lineage and patient age, gender, main site of disease and drug resistance were explored by χ^2^ and Fisher’s exact tests using SPSS 21.0 software (IBM, USA). A p-value of less than 0.05 was considered statistically significant. Ethics clearance was provided by Human Research Ethics Committee of the University of Sydney (project number 2013/126).

## Results

**Figure 1:**
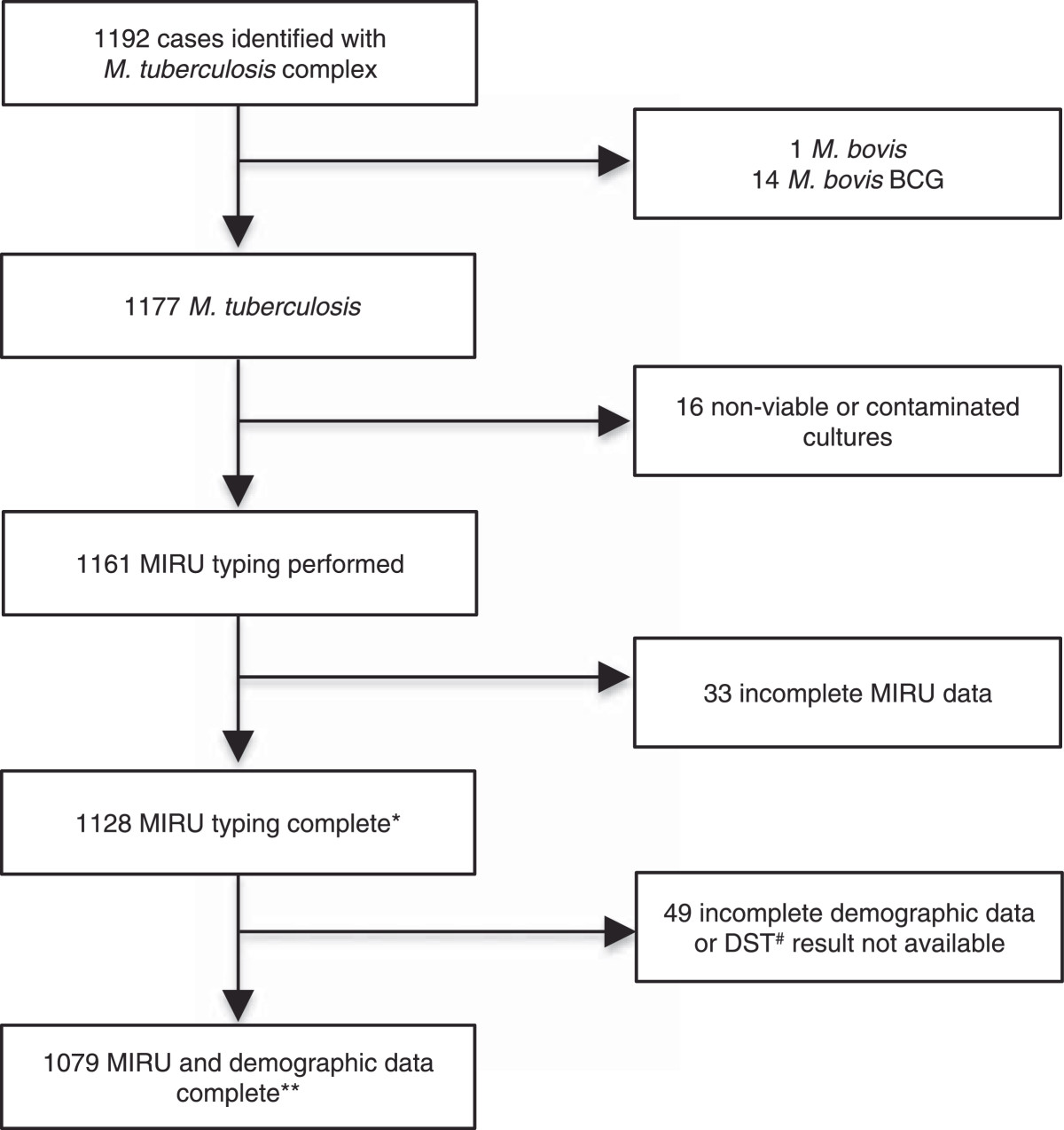
**Flow diagram of new cases with culture-confirmed** ***M. tuberculosis*** **complex diagnosed in NSW.** *Used for population structure and clustering analysis (Table 3) **Used for demographic and drug resistance analysis (Tables 1 and 2) ^#^Phenotypic drug susceptibility test (routinely done for INH and RIF).

A total of 1192 patients were identified with culture-confirmed *M. tuberculosis* complex between January 2010 and December 2012; 1177 with *M. tuberculosis*, 1 with *M. bovis* and 14 with *M. bovi*s BCG. (Figure 1) Of those with *M. tuberculosis,* 1128 (95.8%) were successfully strain typed and included in population structure comparisons and cluster analysis. Demographic analysis was restricted to 1079 (91.7%) cases with complete MIRU typing, DST results and relevant demographic data.

**Figure 2:**
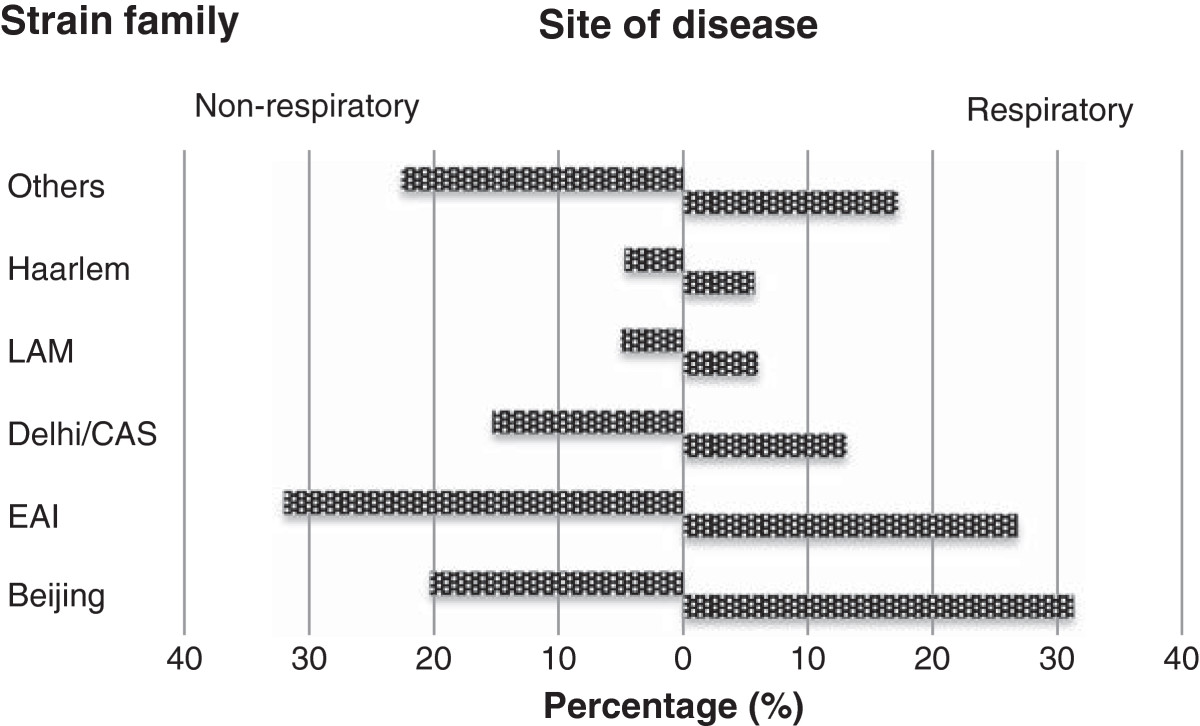
***M. tuberculosis*** **strain family and site of disease.**

**Figure 3:**
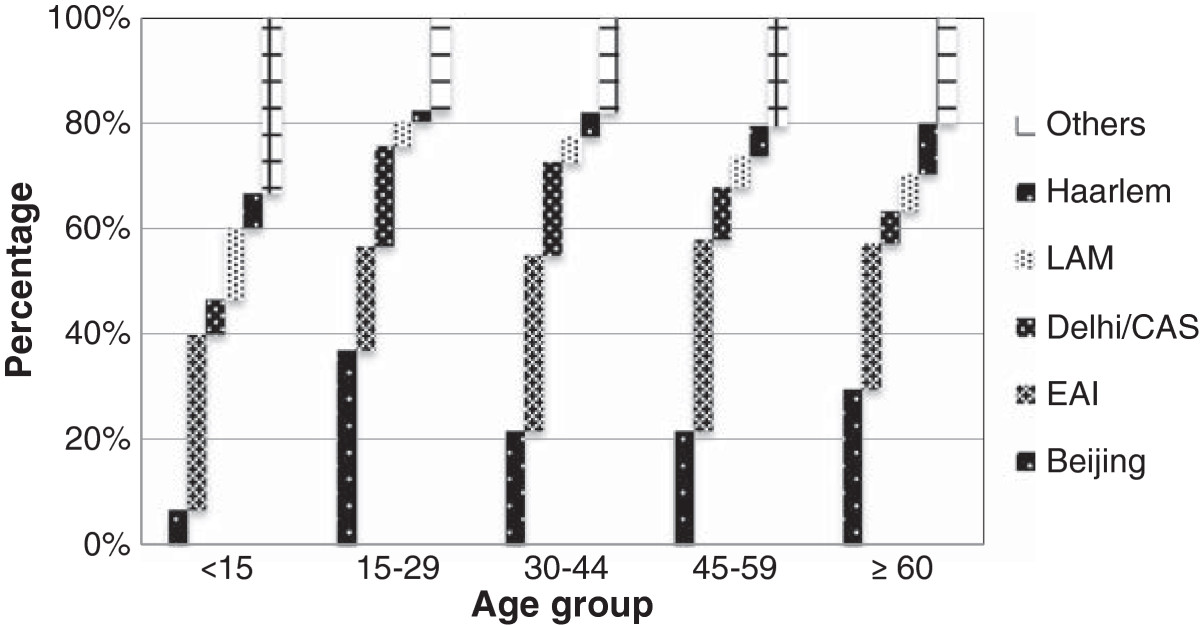
**Relative abundance of prevalent** ***M. tuberculosis*** **strain families in particular age groups.**

Patient demographics, site of disease, strain family and drug resistance profiles are summarized in Table 1. Total case numbers showed little variability, ranging from 351 to 371 between 2010 and 2012. Only 15 (1.4%) children were diagnosed with culture-confirmed tuberculosis, representing a wide variety of lineages; East African Indian (EAI) 5, Latin American Mediterranean (LAM) 2, Beijing 1, Delhi/CAS 1, Haarlem 1, and others 5. A large proportion of cases (270 or 25.0%) were older than 60 years of age. In total 18 cases (1.7%) of multi-drug resistant (MDR) tuberculosis (MDRTB) were detected; 1 being extensively-drug resistant (XDR). Beijing and EAI strain families were most common, accounting for 27.6% and 28.5% of strains, respectively. Beijing was the only strain family significantly associated with respiratory disease and drug resistance (p = 0.02 and p = 0.04, respectively; compared to all other strains). Inversely EAI strain lineage was associated with non-respiratory tuberculosis (p = 0.03) (Figure 2). Mean patient age was significantly associated with lineage (p < 0.001); Delhi/CAS with younger (37 years) and Haarlem with older (57 years) mean age. Beijing family strains were more prevalent among young adults (15-29 years) and the elderly (≥60 years) (p < 0.001) (Figure 3).

**Table 1:** **Demographics, site of disease, strain family and drug resistance profile in new patients with culture–confirmed tuberculosis diagnosed in New South Wales, Australia**

Category	Year			Total
	2010	2011	2012	n (%)
**Gender**				
Male	219 (61.3)	219 (59.0)	188 (53.6)	626 (58.0)
**Age group**				
<15 years	4 (1.1)	5 (1.3)	6 (1.7)	15 (1.4)
15-29 years	99 (27.7)	94 (25.3)	102 (29.1)	295 (27.3)
30-44 years	110 (30.8)	116 (31.3)	102 (29.1)	328 (30.4)
45-59 years	63 (17.6)	60 (16.2)	48 (13.7)	171 (15.8)
≥60 years	81 (22.7)	96 (25.9)	93 (26.5)	270 (25.0)
**Site of disease**				
Respiratory	258 (72.3)	260 (70.1)	241 (68.7)	759 (70.3)
Non-respiratory	99 (27.7)	111 (29.9)	110 (31.3)	320 (29.7)
**Strain family**				
Beijing	93 (26.1)	108 (29.1)	97 (27.6)	298 (27.6)
East African Indian	112 (31.4)	101 (27.2)	95 (27.1)	308 (28.5)
Delhi/CAS	47 (13.2)	53 (14.3)	49 (14.0)	149 (13.8)
LAM	25 (7.0)	14 (3.8)	22 (6.3)	61 (5.7)
Haarlem	13 (3.6)	21 (5.7)	24 (6.8)	58 (5.4)
Other	67 (18.8)	74 (19.9)	64 (18.2)	205 (19.0)
**Drug resistance**				
Isoniazid mono-resistance	21 (5.9)	23 (6.2)	29 (8.3)	73 (6.8)
Multi- or extremely drug resistant (M/XDR)	7 (2.0)	6 (1.6)	5 (1.4)	18 (1.7)
**Total**	**357 (100)**	**371 (100)**	**351 (100)**	**1079 (100)**

Recent trends in the *M. tuberculosis* population structure are documented in Table 2. Both surveys (the 2006-2008 and 2010-2012) included more than 90% of all new cases with culture-confirmed tuberculosis. The proportion of Beijing strains remained stable (24.1% and 27.6%), but EAI strains increased from 11.8% to 28.5%, rivaling Beijing as the dominant strain family in the most populous state of Australia. The relative number of Delhi/CAS strains also doubled from 6.5% to 13.8%. Rates of drug resistance remain similar across both time periods; 6.9% vs 6.8% isoniazid resistance and 1.1% vs 1.7% MDR resistance (resistant to INH and RIF). No cases of RIF mono-resistance were detected during the study period.

**Table 2:** **Changes in** ***M. tuberculosis*** **population structure and drug resistance profiles in New South Wales, Australia**

Characteristic	2006-2008*	2010-2012**	p-value
	n (%)	n (%)	
**Number of isolates included**	855 (100)	1079 (100)	
**Strain family (%)**			
Beijing	206 (24.1)	298 (27.6)	0.58
East African Indian (EAI)	101 (11.8)	308 (28.5)	<0.01
Delhi/CAS	55 (6.5)	149 (13.8)	0.13
**Drug resistance (%)**			
Isoniazid mono-resistance	59 (6.9)	73 (6.8)	1
Multi-drug resistant (MDR)***	9 (1.1)	18 (1.7)	0.56

Notes:

*MIRU-12 typing and spoligotyping.

**MIRU-24 typing.

***Single extensively drug resistant (XDR) case detected in 2010.

Among 1128 cases, 12-loci MIRU detected 572 different profiles with 61.6% (695) of isolates grouped into 139 clusters. The improved resolution provided by 24-loci MIRU detected 983 different profiles with 20.1% (227) of isolates grouped into 82 clusters.(Table 3) The decrease in clustering documented with 24- compared to 12-loci MIRU was most pronounced for EAI and Delhi/CAS strains; clustering rates reduced from 58.6% to 13.6% and 43.7% to 7.6%, respectively. The calculated transmission rate, comparing 24- to 12-loci MIRU, was reduced from 49.3% to 12.8%. There was no evidence of local transmission of MDRTB strains; MIRU-24 profiles of all MDRTB strains were unique. With 12-loci MIRU 17/73 (23.3%) INH-monoresistant cases grouped into 2 clusters, but with 24-loci MIRU only a single 2-member cluster (2/73; 2.7%) was detected.

**Table 3:** **Comparison of** ***M. tuberculosis*** **clustering based on MIRU-12 and MIRU-24 typing**

Characteristic	Dominant strain families						Total	
	Beijing		EAI**		Delhi/CAS		(N = 1128)	
	(N = 309)		(N = 324)		(N = 158)			
MIRU number of loci*	**12**	**24**	**12**	**24**	**12**	**24**	**12**	**24**
Number of clusters	32	42	35	18	17	5	139	82
Number of clustered isolates^#^	249	125	190	44	69	12	695	227
% clustering	80.6	40.5	58.6	13.6	43.7	7.6	61.6	20.1
Average cluster size	8	3	5.4	2.4	4.1	2.8	5	2.8
Transmission rate (%)^##^	70.2	26.9	47.8	8.0	32.9	4.4	49.3	12.8

*MIRU - mycobacterial interspersed repetitive unit.

**EAI – East African Indian strain family (Lineage 3).

^#^Two or more strains with identical MIRU profiles were considered a cluster.

^##^Calculated as (number of clustered isolates - number of clusters)/total number of isolates.

Beijing family strains showed the higher clustering rates; 80.6% with 12- and 40.5% with 24-loci MIRU respectively. The most common 24-loci MIRU profile belonged to a Beijing cluster with 11 members. Cases of tuberculosis associated with the cluster were widely dispersed within the greater Sydney area without evidence of any social contact (see Additional file 1).

Recent report suggested that a subgroup of Beijing family with 12-loci MIRU 223325173533 profile have been associated with increased transmissibility and drug resistance in China [12]. We have identified 94 isolates with this profile; 35, 30 and 29 strains detected in 2010, 2011 and 2012, respectively and 11/94 (11.7%) strains were resistant to INH and 1 isolate was MDR.

## Discussion

We found that Beijing and EAI strains accounted for nearly 60% of all *M. tuberculosis* isolates identified during the study period. While the relative frequency of infections due to Beijing strains has remained stable since 2006 [10], the number of patients infected with EAI and Delhi/CAS strains has doubled in recent years. The dominance of these strain families and recent changes in relative frequency likely reflect changes in immigration patterns in Australia the last decades. Due to the geo-ethnical restriction of major strain families, strain family prevalence is greatly influenced by country of origin. This has been observed in countries such as the USA, the United Kingdom and Canada [13–15]. EAI strains are most abundant in the Indian subcontinent and in East Africa, whereas Beijing strains originated in Asia and are most prevalent in countries such as China and Vietnam [16]. During 2009 88% of tuberculosis cases identified in Australia occurred in people born overseas, with 38% of cases born in India and Vietnam [4], where Delhi/CAS, EAI and Beijing are recognized as predominant strain families [17, 18]. Studies conducted in Cambodia, Indonesia and Papua New Guinea, reported a high prevalence of the same strain families [19–21].

Interestingly, in our study Beijing strains were most common in young adults (15-29 years of age) and in the elderly (≥60 years of age). Its association with younger age has been regarded as a marker of strain emergence in Vietnam [18], but the age associations observed in NSW likely reflect an immigrant cohort effect. The association between Beijing strain family and respiratory tuberculosis requires cautious interpretation, since it included pleural tuberculosis and is not necessarily related to transmission risk. Beijing family strains have been associated with drug resistance in various parts of the world [22–24], although this is not a consistent finding [25]. The number of cases of MDRTB in our study was small and rates of drug resistant disease unchanged since 2006, however, drug resistance was found to be more common in Beijing strains. A retrospective study from Canada also demonstrated an increased frequency of drug resistance among Beijing strains, but found no correlation with the presence of lung cavities, high bacillary loads or severe forms of disease [23]. In Russia, drug resistant Beijing strains spread extensively throughout the prison system [26]. Initial descriptions involved modern Beijing strains, but it has now been recognized that ancient (atypical) Beijing strains are associated with clonal spread of extensive drug resistance in South Africa and Japan [27, 28]. Our research showed that the EAI lineages trains were more common in patients with non-respiratory tuberculosis. Similar findings have previously been reported in the USA after adjusting for confounding factors [29]. Studies have also suggested that EAI is less transmissible and thereby less frequently clustered than Beijing lineage strains [30]. In our study the cluster rate for EAI (13.6%) was three-times lower than that for Beijing lineage strains (40.5%).

Traditional strain clustering indices provide an important marker of transmission within communities and have been used to analyze transmission patterns and guide public health intervention. However, its value may be reduced in areas dominated by imported disease, where identified clusters are less likely to be epidemiologically linked [31]. The problem can be more pronounced when strain typing methods have sub-optimal discriminatory power. Compared to 12-loci, 24-loci MIRU typing provides enhanced resolution, but this remained sub-optimal especially for Beijing strains, as reflected by the high clustering rate (40.5%) and wide geographic dispersal observed among the largest single cluster, without evidence of epidemiological links. Studies from Asia and Russia have emphasized high levels of genome homoplasy within the Beijing strain family [32, 33], and identified the need for more accurate cluster differentiation. Analysis of an additional 4 loci has been suggested to improve Beijing strain family discrimination of standard MIRU-24 [34, 35]. Sub-optimal discrimination is an important limitation of MIRU-24 in areas where Beijing family strains are common, since it limits the ability to identify transmission chains and direct public health responses. High resolution typing methods based on whole genome sequencing showed the promise for more targeted public health responses in non-endemic areas dominated by imported disease [36].

Some limitations of our study should be acknowledged. First, our dataset did not include all tuberculosis cases, although the 1079 cases included in the study represent 71% of tuberculosis notifications in NSW during the study period. Second, since this was a laboratory-based study we did not have access to detailed clinical or epidemiological data. Third, some strain families eg. Euro-American lineages with unique 24-loci MIRU patterns could not be clearly distinguished from the MIRU-VNTR*plus* database. The previous study (2006-2008), to which our data was compared, used a combination of 12-loci MIRU and spoligotyping for strain family assignment, leading to some minor discrepancies that had to be resolved. However, only 24 isolates were affected. Thus we believe that these limitations would not have influenced the direction of our conclusions or statistical significance of our findings. The calculated transmission rate should be interpreted with caution, given the high proportion of Beijing isolates among clustered strains (125/227; 55%) and the typing method limitations discussed earlier. Geographic case distribution and contact information suggests that the calculated transmission rate could overestimate local transmission.

## Conclusion

As in other low-incidence countries the *M. tuberculosis* population structure in Australia is shaped by migrant flows. Temporal dynamics were characterized by a relative increase in Indo-Oceanic and EAI global lineages among patients with culture-confirmed tuberculosis in New South Wales, especially among young children. Standard 24-loci MIRU typing provided sub-optimal strain differentiation and cluster identification. High resolution typing methods are required for accurate transmission tracking, especially of Beijing family strains, to help guide public health responses.

## Electronic supplementary material

Additional file 1: Geomapping of 11-member Beijing cluster identified by 24-loci MIRU in New South Wales, Australia (2010-2012).(PDF 2 MB)

Below are the links to the authors’ original submitted files for images.

Authors’ original file for figure 1

Authors’ original file for figure 2

Authors’ original file for figure 3
