# Supplementary material for: Temporal dynamics of Mycobacterium tuberculosis genotypes in New South Wales, Australia
Source: BMC Infect Dis. 2014 Aug 23;14:455. doi: 10.1186/1471-2334-14-455 (PMC4262242; doi:10.1186/1471-2334-14-455)

Supplementary document

Geomapping of 11-member Beijing cluster identified by 24-loci MIRU in New South Wales, Australia (2010-2012)

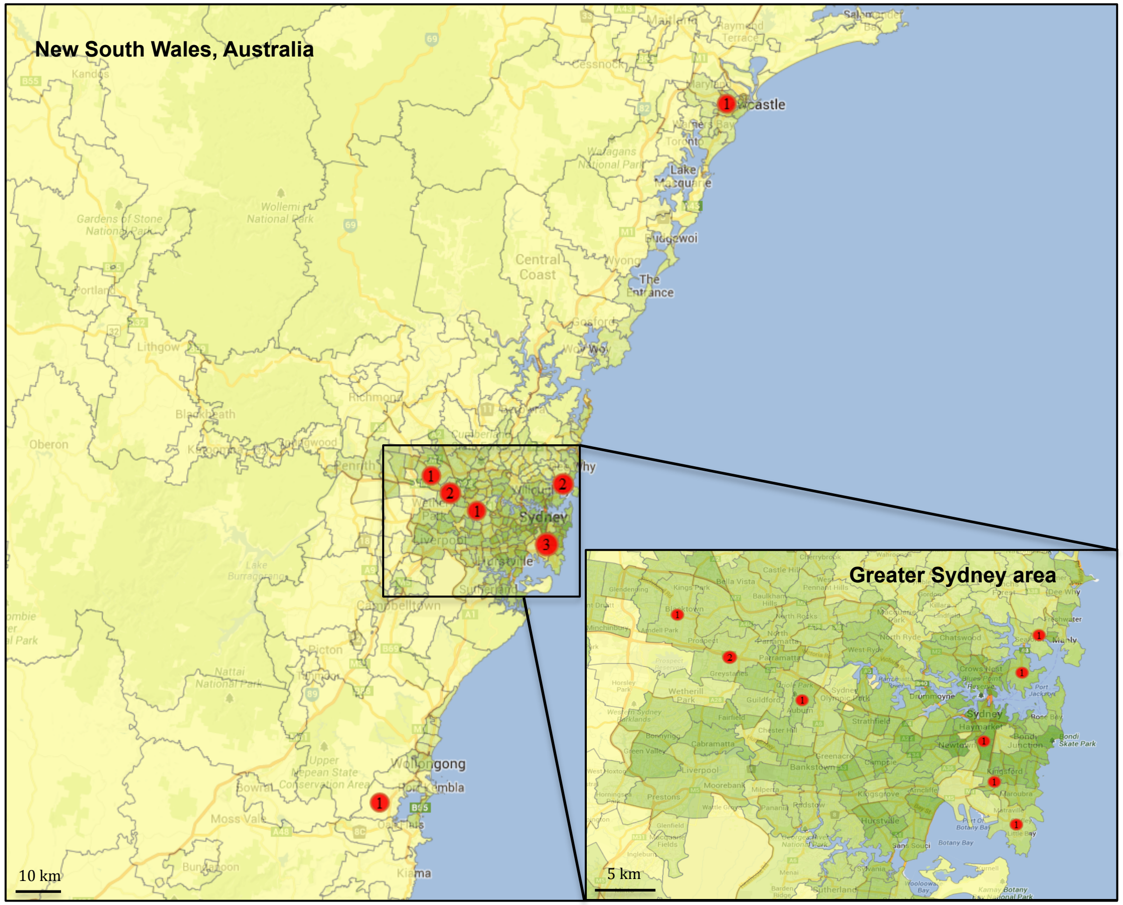

Supplement: Supplementary file 1 — Additional file 1: Geomapping of 11-member Beijing cluster identified by 24-loci MIRU in New South Wales, Australia (2010-2012).(PDF 2 MB) [file 12879_2014_4047_MOESM1_ESM.pdf]
