# Supplementary figures and images for: Temporal dynamics of Mycobacterium tuberculosis genotypes in New South Wales, Australia
Source: BMC Infect Dis. 2014 Aug 23;14:455. doi: 10.1186/1471-2334-14-455 (PMC4262242; doi:10.1186/1471-2334-14-455)

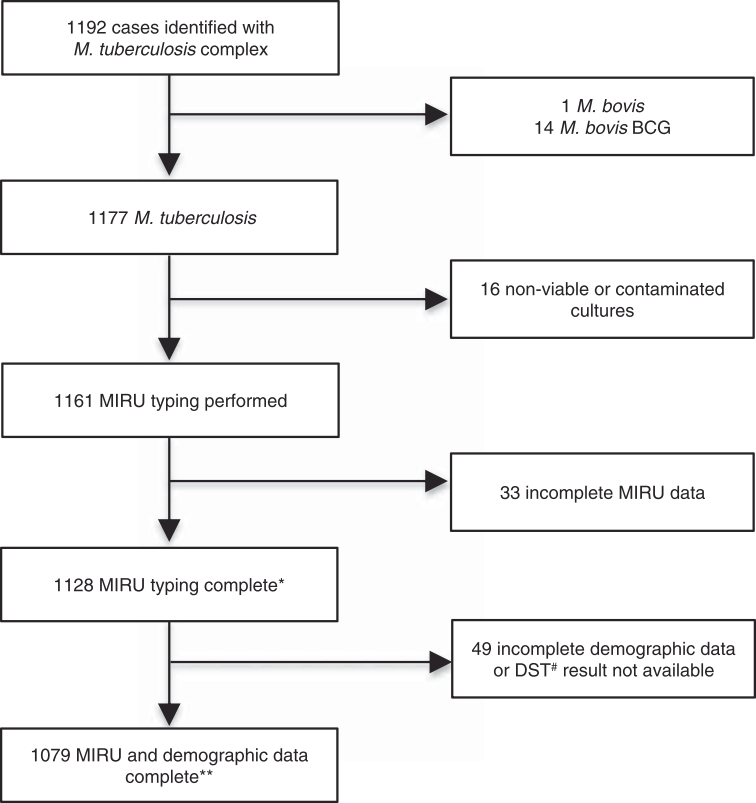

Supplement: Supplementary file 2 — Authors’ original file for figure 1 [file 12879_2014_4047_MOESM2_ESM.pdf]

**Strain family**

**Site of disease**

Non-respiratory

Respiratory

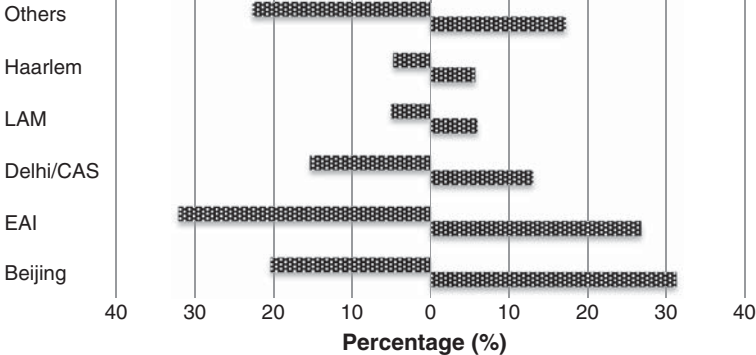

Supplement: Supplementary file 3 — Authors’ original file for figure 2 [file 12879_2014_4047_MOESM3_ESM.pdf]

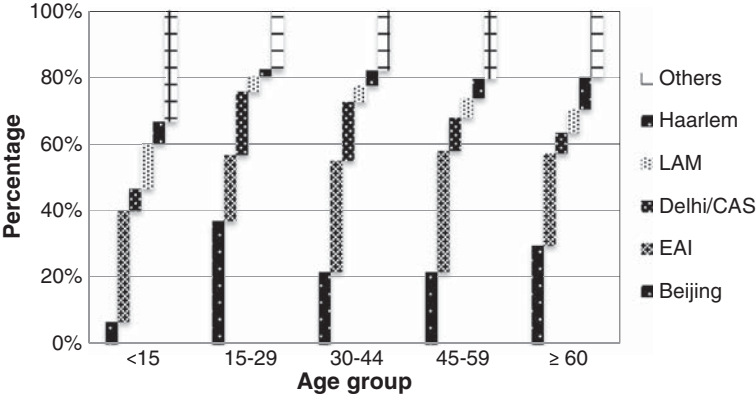

Supplement: Supplementary file 4 — Authors’ original file for figure 3 [file 12879_2014_4047_MOESM4_ESM.pdf]
